# Exclusive breastfeeding: Measurement to match the global recommendation

**DOI:** 10.1111/mcn.13409

**Published:** 2022-08-23

**Authors:** Silvia Alayón, Veronica Varela, Altrena Mukuria‐Ashe, Jeniece Alvey, Erin Milner, Sarah Pedersen, Jennifer Yourkavitch

**Affiliations:** ^1^ USAID Advancing Nutrition Arlington Virginia USA; ^2^ Save the Children US Washington District of Columbia USA; ^3^ John Snow, Inc. Arlington Virginia USA; ^4^ Public Health Institute/USAID Global Health Technical Professionals Washington District of Columbia USA; ^5^ Public Health Institute/USAID Sustaining Technical and Analytical Resources Washington District of Columbia USA; ^6^ Credence/USAID Global Health Technical Professionals Washington District of Columbia USA; ^7^ Results for Development Washington District of Columbia USA

**Keywords:** breastfeeding, breastfeeding duration, infant and child nutrition, infant feeding, measurement, monitoring and evaluation, survey methods

## Abstract

The World Health Organization (WHO) and United Nations Children's Fund (UNICEF) recommend exclusive breastfeeding (EBF) for the first 6 months of life. To estimate the proportion of infants that are exclusively breastfed, many agencies use the point prevalence of EBF among infants currently 0–5.9 months of age, as recommended by WHO and UNICEF. This measure tends to overestimate the percentage of infants that are exclusively breastfed for the entire recommended period. We compared five methods of measuring EBF, using data from three large‐scale cross‐sectional surveys. The five methods were: the WHO/UNICEF recommended method (EBF‐24H); an estimate of EBF for 6 months, using the 24‐h recall among infants 4–5.9 and 6–7.9 months (EBF‐24H‐Pul); a since birth recall (EBF‐SB); an estimate of EBF for 6 months, using the since‐birth recall among infants 4–5.9 and 6–7.9 months (EBF‐SB‐Pul); a retrospective measure of EBF collected from infants 6–11.9 months, based on the age of introduction of liquids and foods (EBF‐AI). EBF‐24H‐Pul and EBF‐SB‐Pul produced lower estimates of EBF than other measures, while also aligning better with the WHO recommendation, but may be difficult to estimate from multipurpose surveys due to sample size limitations. The EBF‐AI method produced estimates between these, aligns well with the WHO recommendation and can be easily collected in large‐scale household surveys. Additional validation of the EBF‐24‐Pul, EBF‐SB‐Pul, and EBF‐AI methods is recommended to understand how accurately they measure EBF for the recommended 6‐month period.

## Key points

- Exclusive breastfeeding infants for the first 6 months of life is recommended by the World Health Organization and UNICEF for the benefits it provides to both the infant and the mother.
- The measure currently recommended for global monitoring of exclusive breastfeeding is the prevalence of exclusive breastfeeding among infants less than 6 months, based on a 24‐h recall.
- Though this measure is easy to collect in large‐scale household surveys, it is often misinterpreted as the percent of infants who are fed according to the recommendation, resulting in an overestimation.
- Other survey‐derived measures of exclusive breastfeeding are available, for example, such as calculating the midpoint prevalence among infants 4–5.9 and 6–7.9 months, a method developed by Pullum and a since birth recall among infants aged 6–11.9 months of age.
- These are similarly feasible to collect in household surveys and align better with the practice of exclusive breastfeeding for 6 months as recommended by the WHO.

## Graphical abstract

To estimate the proportion of infants that are exclusively breastfed, many agencies use the point prevalence of exclusive breastfeeding (EBF) among infants currently 0–5.9 months of age, as recommended by World Health Organization (WHO) and UNICEF. This measure tends to overestimate the percentage of infants that are exclusively breastfed for the entire recommended period. We compared five methods of measuring EBF, using data from three large‐scale cross‐sectional surveys.

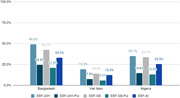

## 1 INTRODUCTION

Optimal breastfeeding is critical for the health and development of infants and young children, and for women's health. Children who are breastfed for longer periods have lower infectious morbidity, fewer dental malocclusions, and higher intelligence than children who are breastfed for shorter periods or are not breastfed at all (Victora et al., 2016). Breastfeeding has also been shown to benefit mothers by protecting against breast cancer, diabetes, ovarian cancer, and improving birth spacing (Victora et al., 2016). The World Health Organization (WHO) defines exclusive breastfeeding as an infant receiving no other food or drink, not even water, except breast milk (including milk expressed or from a wet nurse), while allowing for ingestion of prescribed oral rehydration solutions, drops, and syrups such as vitamins, minerals, and medicines (WHO & UNICEF, 2021; World Health Assembly, 2001). Infants under 6 months of age who are not exclusively breastfed are at significantly higher risk of all‐cause mortality and infection‐related mortality compared to exclusively breastfed infants (Sankar et al., 2015). Prelacteal feeding, that is feeding an infant anything other than breast milk in the first 3 days of life, is associated with higher morbidity and mortality in the first year of life and also earlier cessation of breastfeeding (Nguyen et al., 2020; Pérez‐Escamilla et al., 2022). Acknowledging the benefits, the WHO and United Nations Children's Fund (UNICEF) recommend initiation of breastfeeding within the first hour of birth, exclusive breastfeeding for the first 6 months of life, and continued breastfeeding until 2 years of age or beyond (WHO & UNICEF, 2003). Despite these recommendations, worldwide only about 44% of infants under 6 months are exclusively breastfed (WHO, 2021).

To estimate the proportion of infants that are exclusively breastfed, many agencies follow the recommendation of the WHO and UNICEF and use the point prevalence of exclusive breastfeeding among infants currently 0–5.9 months of age, defined as the proportion of infants currently 0–5.9 months that were exclusively breastfeeding in the previous 24 h (WHO & UNICEF, 2021). Calculation of this indicator relies on mothers’ reports of what the infant consumed in the 24 h before the survey (WHO & UNICEF, 2021). This method, employed in many household surveys such as the Demographic and Health Surveys (DHS) and UNICEF Multiple Indicator Cluster Surveys (MICS) in addition to programme monitoring and evaluation assessments, is used to report the prevalence of exclusive breastfeeding in a country or region (ICF, 2020; UNICEF, 2020).

Despite the clear definition of exclusive breastfeeding provided by the WHO, there is a discrepancy between the recommendation and how exclusive breastfeeding is typically measured and reported. Measuring the point prevalence of exclusive breastfeeding in the previous 24 h tends to overestimate the percentage of infants that are exclusively breastfed for the entire recommended period (Pullum, 2014). The use of point prevalence of exclusive breastfeeding among 0–5.9 month olds as a proxy for 6 months of exclusive breastfeeding remains recommended and widespread, though it is often misinterpreted or miscommunicated as the proportion of infants who did exclusively breastfeed for a full 6 months.

This is a challenge for other health behaviours that require sustained practice. For example, recent recommendations for evaluating the effectiveness of smoking cessation programmes include measuring sustained abstinence after discharge for 3‐ and 6‐month periods, not just point prevalence (Piper et al., 2020).

One alternative to the 24‐h recall is a since‐birth recall, which asks mothers to recall if certain foods and liquids were introduced to infants since birth, which is typically collected among children 0–5.9 months (Fenta et al., 2017; Van Beusekom et al., 2013). If a food or liquid was introduced at any time before the survey, the infant would be classified as nonexclusively breastfed for the recommended 6‐month period. Another option is to ask mothers of infants 6–11.9 months of age to recall their feeding practices, specifically the age at which foods and liquids were introduced, which would not be limited by the age distribution of respondents and allows us to capture the full eligibility period. A third alternative is to use the midpoint between the prevalence of exclusive breastfeeding among 4–5.9 month olds and the prevalence of exclusive breastfeeding among 6–7.9 month olds (Pullum, 2014; Rutstein & Rojas, 2006).

The 24‐h recall carries with it an implicit assumption that the way infants are breastfed on the day before the survey is consistent with how they were fed from birth to that date and will continue until they are at least 6 months old (Pullum, 2014). A since‐birth recall collected among infants 0−5.9 months of age also assumes consistent continued feeding for the remainder of the eligibility period, while assessing exclusive breastfeeding after 6 months of age relies on a long period of maternal recall.

The purpose of this article is to examine measurement‐dependent differences for exclusive breastfeeding among populations in three countries and discuss how the measurement methods align with the WHO recommendation of exclusive breastfeeding for the first 6 months of life.

## 2 METHODOLOGY

We compared five methods of measuring exclusive breastfeeding using data from three large‐scale household surveys. The five methods include:

1. Prevalence of exclusive breastfeeding among infants less than 6 months, based on a 24‐h recall (EBF‐24H).
2. Percentage of infants who were exclusively breastfed for the recommended first 6 months, based on a 24‐h recall, using the midpoint between EBF‐24H among infants 4–5.9 and 6–7.9 months old (EBF‐24H‐Pul).
3. Percentage of infants less than 6 months who were not given anything other than breast milk since birth (exclusive breastfeeding since birth among infants less than 6 months of age) (EBF‐SB).
4. Percentage of infants who were exclusively breastfed for the recommended first 6 months of age based on a since‐birth recall, using the midpoint EBF‐SB between infants 4–5.9 and 6–7.9 months old (EBF‐SB‐Pul).
5. Percentage of infants 6–11.9 months who did not consume anything other than breast milk for their first 6 months of life (exclusive breastfeeding for 6 months noncensored) (EBF‐AI).

Though the convention is to refer to infants who have not yet completed their 6th month as ʻ5 months old’, in this article, we refer to children less than 6 months old as ʻ0–5.9 months’. In this way, we eliminate ambiguity about the inclusion of infants who had already completed their 5th month, but not their 6th month (i.e., infants whose age is greater than 5 completed months, but less than 6 completed months).

We used data from three cross‐sectional surveys conducted by the Alive & Thrive initiative. The datasets comprised baseline data collected in 2010 in Bangladesh and Viet Nam, and in 2017 in Nigeria. The surveys included 20 upazilas (subdistricts) in Bangladesh, 40 communes in Viet Nam and 39 local government areas in two states of Nigeria—Kaduna and Lagos (Flax, 2019; Menon et al., 2016). Within each upazila in Bangladesh, five unions were randomly selected. Unions are an administrative unit one level below the upazila and consist of several villages (National Institute of Population Research and Training (NIPORT) & ICF, 2020). Within each union, two villages were randomly selected for a total of 200 villages (Menon et al., 2016). In both Bangladesh and Viet Nam, a household census was conducted to identify households with eligible infants; from this census, households were selected using systematic sampling beginning with a random seed point until the desired sample size was reached (Menon et al., 2016). In Nigeria, a gridded population sampling (i.e., geo sampling) was complemented by a random walk method to obtain the desired sample size for infants 0–5.9 months (V. Flax, personal communication, September 10, 2021). All data sets used in this analysis are publicly available (Flax, 2019; International Food Policy Research Institute IFPRI, 2020a, 2020b). The surveys included comparison and intervention areas, which were part of the Alive & Thrive initiative, but the survey occurred before interventions were implemented. Each survey included data on current feeding practices of infants 0–5.9 months of age, first asking about feeding in the 24 h before the survey and then asking mothers at what age the infant was first introduced to several foods and beverages. Retrospective data on the age of introduction of liquids and solid or semi‐solid foods among infants 6–23 months of age were also collected in each country, though we limited our analysis to the infants 6–11.9 months of age.

Five different methods of estimating exclusive breastfeeding are explored in this article:

1) Prevalence of exclusive breastfeeding among infants less than 6 months, based on a 24‐h recall (EBF‐24H):
$$\frac{\text{Number}\;\text{of}\;\text{infants}\;0–5.9\;\text{months}\;\text{of}\;\text{age}\;\text{who}\;\text{received}\;\text{only}\;\text{breast}\;\text{milk}\;\text{during}\;\text{the}\;\text{previous}\;\text{day}}{\text{Number}\;\text{of}\;\text{infants}\;0–5.9\;\text{months}\;\text{of}\;\text{age}}.$$
  An infant under 6 months of age was considered to be exclusively breastfeeding under this indicator if in the previous 24 h of the survey interview the index child was given breast milk and no other food or liquid.
2) Percentage of infants who were exclusively breastfed for the recommended first 6 months, based on a 24‐h recall (EBF‐24H‐Pul):
  First proposed by Pullum (2014), this method uses the same set of questions that are used to calculate EBF‐24H above. Infants are classified as exclusively breastfeeding according to the same definition as in EBF‐24H above. This measure uses the smoothed prevalence of exclusive breastfeeding, calculated separately for each 2‐month age group between 0 and 7.9 months. The average, weighted by sample size, between the smoothed prevalence calculations for of infants aged 4–5.9 and those aged 6–7.9 months is used to estimate the percentage of infants exclusively breastfeeding at the midpoint of the two groups, which is 6 months (Pullum, 2014; Rutstein & Rojas, 2006).
3) Percentage of infants less than 6 months who were not given anything other than breast milk since birth (exclusive breastfeeding since birth among infants less than 6 months of age) (EBF‐SB):
$$\frac{\text{Number}\;\text{of}\;\text{infants}\;0–5.9\;\text{months}\;\text{of}\;\text{age}\;\text{who}\;\text{have}\;\text{not}\;\text{been}\;\text{given}\;\text{any}\;\text{liquids}\;\text{or}\;\text{foods}\left(\text{other}\;\text{than}\;\text{breast}\;\text{milk}\right)\text{since}\;\text{birth}}{\text{Number}\;\text{of}\;\text{infants}\;0–5.9\;\text{months}\;\text{of}\;\text{age}}.$$
  An infant under 6 months of age was considered to be exclusively breastfeeding if in the previous 24 h before the survey the index child was given breast milk and had not been introduced to any other liquids or foods since birth.
4) Percentage of infants who were exclusively breastfed for the recommended first 6 months of age based on a since‐birth recall (EBF‐SB‐Pul):
  The same methodology was used for this measure as is described for measure number two above (EBF‐24H‐Pul). However, instead of using the 24‐h recall, Pullum's method is applied to the prevalences of exclusive breastfeeding obtained, for the same two age groups, using a since‐birth recall.
5) Percentage of infants 6–11.9 months who did not consume anything other than breast milk for their first 6 months of life (exclusive breastfeeding for 6 months, based on the age of introduction) (EBF‐AI):

$$\frac{\text{Number}\;\text{of}\;\text{children}\;6–11.9\;\text{months}\;\text{who}\;\text{were}\;\text{not}\;\text{given}\;\text{any}\;\text{other}\;\text{liquids}\;\text{or}\;\text{foods},\text{besides}\;\text{breast}\;\text{milk},\text{before}\;\text{the}\;\text{age}\;\text{of}\;\;\text{months}}{\text{Number}\;\text{of}\;\text{children}\;6–11.9\;\text{months}\;\text{of}\;\text{age}}.$$

An infant 6–11.9 months of age was considered exclusively breastfed under this indicator if all foods and liquids other than breast milk were introduced at or after the age of 6 months. Pullum's method is not necessary when using the EBF‐AI estimate as it represents a direct measure of the proportion of infants who were exclusively breastfed for 6 months.

For all five measures, infants who were given vitamin or mineral syrups, medicine, and/or oral rehydration solutions, but no other liquid or solid/semi‐solid food aside from breast milk were included in the numerator.

Finally, we calculated the median duration of exclusive breastfeeding, defined as the age at which 50% or less of infants are exclusively breastfed (Croft et al., 2018). For the EBF‐24H and EBF‐SB indicators, the median estimates were calculated among infants less than 8 months of age as described by Rutstein and Rojas (2006). Infants were divided into 2‐month age groups and for each group a smoothed prevalence was estimated. The mid‐point between the age group for which the prevalence falls below 50% and the age group that precedes it is taken as the median. This method has been used to report median duration of *any* breastfeeding in Demographic and Health Surveys (Rutstein & Rojas, 2006). Median duration estimates derived from EBF‐24H and EBF‐SB were then compared to the median duration of exclusive breastfeeding using survival curves constructed from the EBF‐AI indicator.

For all three surveys infants less than 12 months were included in the analysis and the total sample sizes for each country were: 1385 (Bangladesh), 1408 (Viet Nam) and 3990 (Nigeria). The total sample for each country was split into two groups: infants less than 6 months and infants 6–11.9 months.

## 3 RESULTS

Table 1 presents demographic information for the respondents in each country. The mean age in months of the infants in the 0–5.9 group was 3.3 in Bangladesh, 3.5 in Viet Nam and 2.9 in Nigeria. Among the older infants (6–11.9 months), the mean age in months was 8.7 in Bangladesh, 8.9 in Viet Nam and 8.9 in Nigeria. Fewer mothers interviewed in Bangladesh and Nigeria worked outside the home compared to the Vietnamese mothers. In Viet Nam, over 85% of the mothers worked outside the home, mostly as farmers, which was also the predominant occupation of the heads of households in Viet Nam. In Bangladesh, the heads of households were mostly manual workers, followed by farmers and working in business, as traders, or self‐employed. In Nigeria, over 50% of household heads worked in business, as traders, or were self‐employed.

**Table 1:** Demographic characteristics of survey respondents in Bangladesh, Viet Nam and Nigeria

	Bangladesh		Viet Nam		Nigeria	
Demographics	Infants 0–5.9 months of age (*n* = 977)	Infants 6–11.9 months of age (*n* = 408)	Infants 0–5.9 months of age (*n* = 948)	Infants 6–11.9 months of age (*n* = 460)	Infants 0–5.9 months of age (*n* = 2433)	Infants 6–11.9 months of age (*n* = 1557)
Mean age in months of infant (standard deviation)	3.3 (1.6)	8.7 (1.8)	3.5 (1.5)	8.9 (1.7)	2.9 (1.7)	8.9 (1.7)
Mother does any work outside of home						
Yes	7.2%	6.6%	89.0%	86.7%	18.6%	17.5%
No	92.8%	93.4%	11.0%	13.3%	81.4%	82.5%
Mother's main occupation						
Farmer	0.0%	0.0%	51.6%	47.4%	3.0%	2.9%
Service/salaried staff	0.8%	1.2%	10.7%	14.1%	4.9%	5.6%
Manual worker	0.7%	1.0%	10.3%	5.7%	0.0%	0.0%
Business/traders/self employment	1.3%	0.5%	12.0%	13.9%	55.9%	61.0%
Household work/housewife	95.6%	96.8%	13.1%	17.2%	30.0%	22.8%
Jobless	0.0%	0.0%	0.0%	0.0%	4.9%	6.2%
Other	1.5%	0.5%	2.3%	1.7%	1.4%	1.5%
Household head's main occupation						
Farmer	21.2%	25.3%	42.6%	41.0%	19.9%	22.4%
Service/salaried staff	14.9%	18.6%	11.1%	14.1%	21.6%	2.0%
Manual worker	32.9%	27.2%	16.1%	13.0%	0.0%	0.0%
Business/traders/self employment	25.0%	22.8%	25.9%	27.1%	54.2%	54.3%
Household work/housewife	0.0%	0.0%	0.1%	0.0%	2.3%	1.0%
Jobless	1.1%	1.2%	0.8%	0.4%	1.0%	0.5%
Other	4.9%	4.9%	3.5%	4.4%	1.0%	1.4%

**Figure 1:**
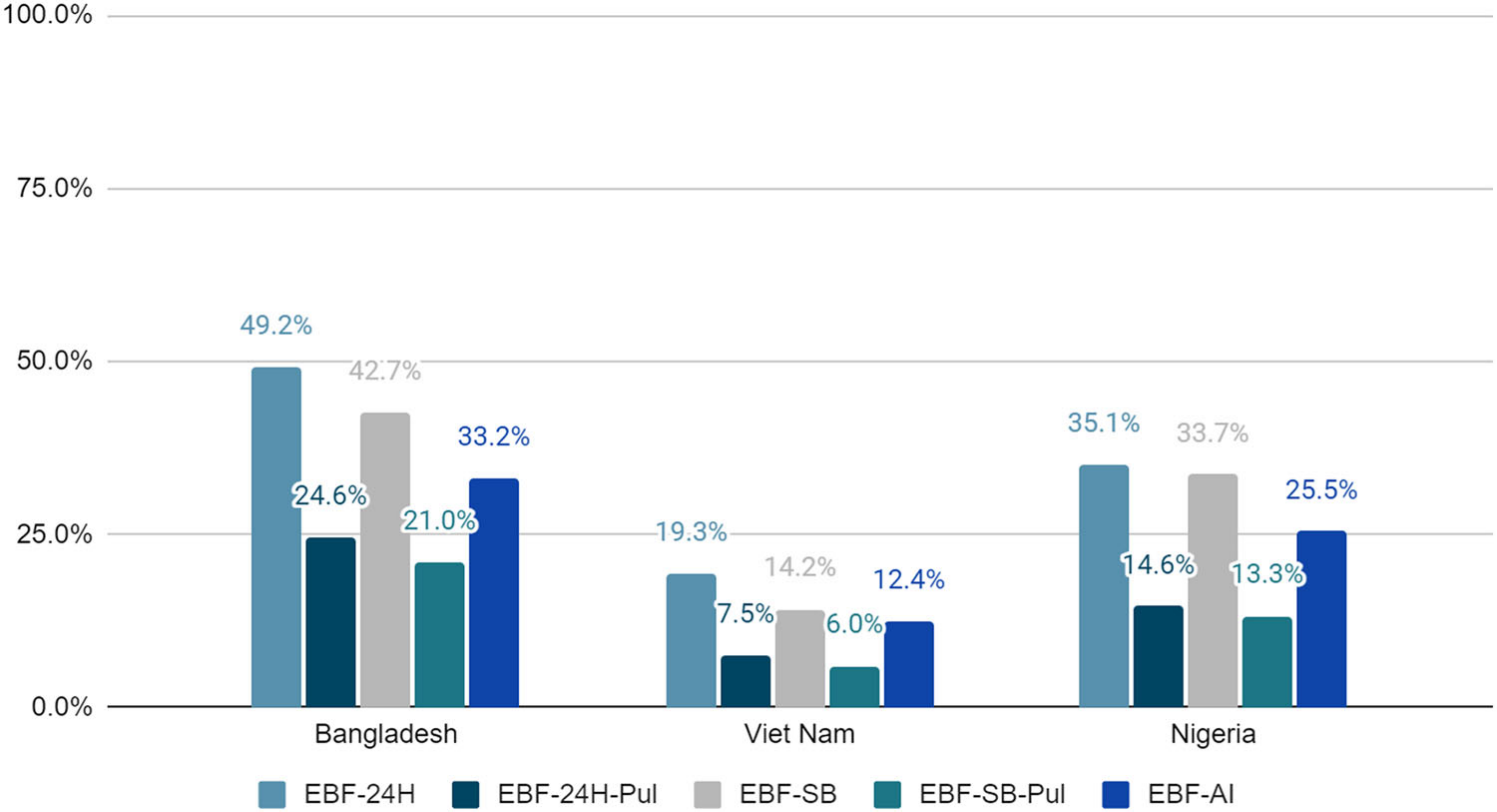
Proportion of infants exclusively breastfed, by method. EBF‐AI, age at introduction; EBF‐SB, since‐birth recall; EBF‐SB‐Pul, Pullum's method applied to a since‐birth recall; EBF‐24H, 24‐hour recall; EBF‐24H‐Pul, Pullum's method applied to a 24‐hour recall

Figure 1 presents the estimated percentage of infants who were exclusively breastfed by survey for each of the five measures described above. In all three surveys, the proportion of infants in the study sample who were exclusively breastfed was below 50%, regardless of the method used. Of the three direct prevalence measures—EBF‐24H, EBF‐SB and EBF‐AI—EBF‐24H produced the highest estimates for all surveys (49.2% in Bangladesh, 19.3% in Viet Nam and 35.1% in Nigeria); the estimate using EBF‐AI was the lowest (33.2% in Bangladesh, 12.4% in Viet Nam and 25.5% in Nigeria). The absolute difference between these two estimates ranged from 6.9 percentage points in Viet Nam to 16.0 percentage points in Bangladesh.

In all three surveys, the prevalence of exclusive breastfeeding until 6 months of age is lower when using Pullum's method applied to the 24‐h recall and the since birth recall (EBF‐24H‐Pul and EBF‐SB‐Pul), compared to the estimates obtained using EBF‐24H, EBF‐SB and EBF‐AI. For example in Bangladesh, 33.2% of infants 6–11.9 months of age were exclusively breastfed for 6 months using the EBF‐AI method. The prevalence of exclusive breastfeeding for 6 months in Bangladesh applying Pullum's method to the EBF‐24 and EBF‐SB indicators results in estimates of 24.6% and 21.0%, respectively.

**Figure 2:**
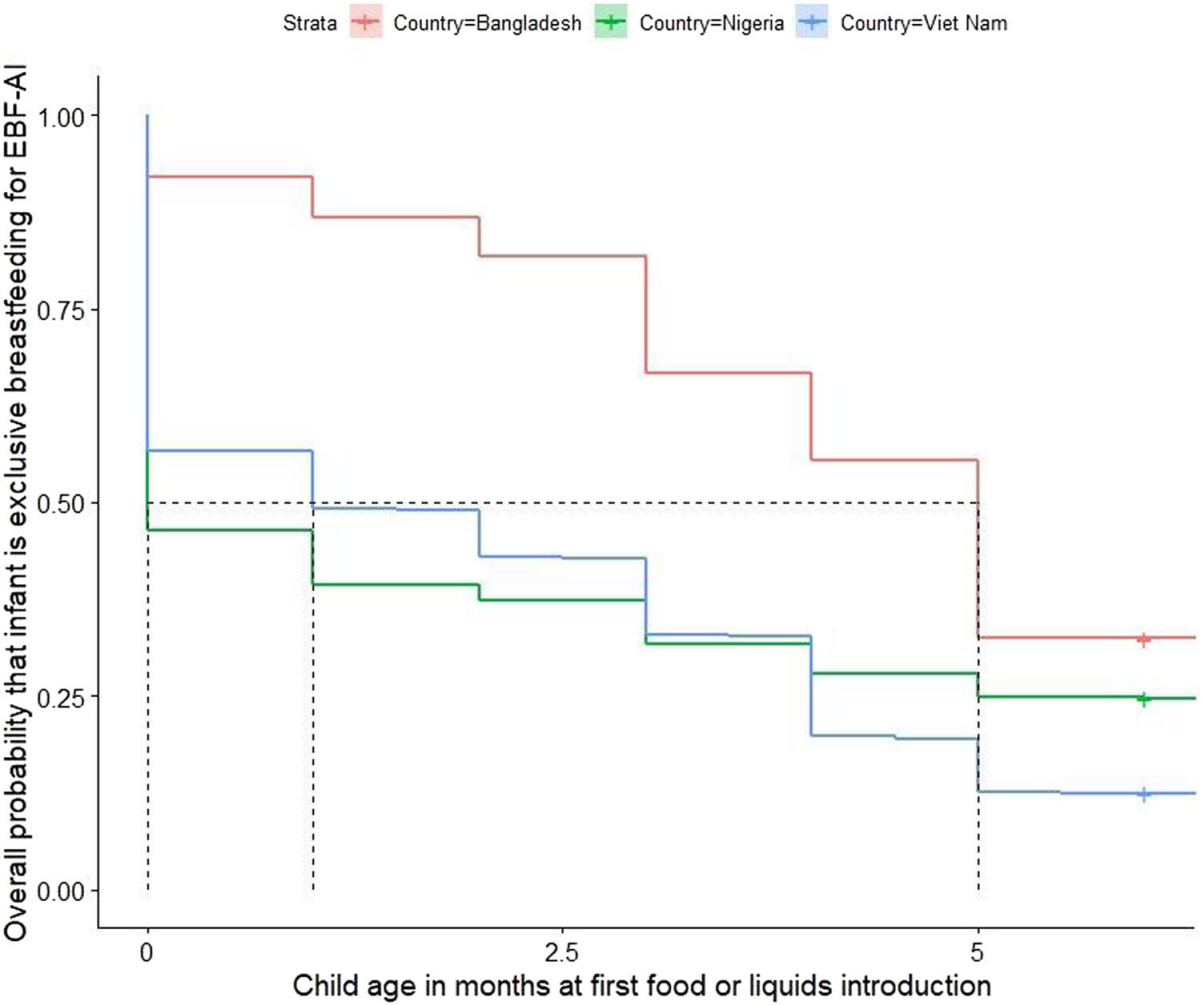
Survival curve of exclusive breastfeeding for EBF‐AI, by survey

We constructed survival curves (Figure 2) to show the time to exclusive breastfeeding cessation and to estimate the median duration of exclusive breastfeeding for the EBF‐AI indicator because it includes data that cover the entire 6‐month period for each infant. Like the area graphs recently added to the WHO and UNICEF infant and young child (IYCF) indicator guide, these can provide useful information about the age at which infants are most at risk of being introduced to foods and liquids other than breast milk (WHO & UNICEF, 2021). In Bangladesh, for example, the survival curve indicates that infants are at highest risk for being introduced to foods or liquids other than breast milk around 3 and 5 months.

## 4 DISCUSSION

In this article, we estimated exclusive breastfeeding prevalence five ways and median time to exclusive breastfeeding cessation three ways to examine measurement‐dependent differences among the same populations in three countries. The EBF‐24H indicator consistently resulted in the highest estimates across all three countries included in this analysis. Several factors result: (1) failure to capture prelacteal feeding; (2) failure to capture intermittent use of complementary feeds; and (3) the assumption that the feeding pattern at the time of the survey will continue until 6 months of age (Fenta et al., 2017; Greiner, 2014; Khanal et al., 2016). EBF‐SB is more sensitive to the first two issues, that is, is able to capture both prelacteal feeding and intermittent use of complementary feeds. Using Pullum's method to estimate the percentage of infants who are breastfed for 6 months by averaging the prevalence among 4–5.9 month olds and 6–7.9 month olds using either EBF‐24H or EBF‐SB produces results lower than the other methods. By shifting the measurement age closer to the 6‐month mark, Pullum's method addresses the third issue above—the assumption that feeding patterns will persist to 6 months of age. Pullum's measure, aligns well with the WHO recommendation; unlike EBF‐24H and EBF‐SB, Pullum's method is able to distinguish between infants who were exclusively breastfed at some point between 0 and 5.9 months of age and those who continued to exclusively breastfeed for a longer period. However, Pullum's method has not been validated and may ʻovercorrect’. For example, if everyone stopped EBF at age 6 months, then Pullum's method would indicate 50% prevalence of EBF at 6 months. However, this method is not likely to be grossly inaccurate in most populations since EBF cessation is variable (as shown in Figure 2), but, notably, it did produce a lower estimate than EBF‐SB.

Like the survival curves constructed for our analysis, the area graphs now recommended by the WHO and UNICEF (2021) will be important to help understand cessation of exclusive breastfeeding. Having an alternative method to study whether infants are exclusively breastfed for the entire recommended time versus at one point in time is useful because it helps understand the proportion of infants that, fed according to the WHO recommendations, receive the full benefits of optimal breastfeeding. Several studies in low‐income settings in Asia, Africa and Central America compared the use of a single 24‐h recall with recall since birth with similar results to ours (Engebretsen et al., 2007; Fenta et al., 2017; Hussein et al., 2019; Khanal et al., 2016; Roberts et al., 2018; Van Beusekom et al., 2013). Fenta and colleagues, for example, compared the use of a single 24‐h recall, seven repeated 24‐h recalls and since‐birth recall in rural Ethiopia (2017). They found that when compared to seven repeated recalls, a single 24‐h recall overestimated exclusive breastfeeding, while recall since birth among infants less than 6 months of age resulted in an estimate of exclusive breastfeeding that was only slightly lower (Fenta et al., 2017). A prospective study in Sweden, collected daily feeding records from infants beginning on the 4th or 8th day of life showed that current status (i.e., exclusively breastfeeding in the previous 24 h) was consistently higher at 2, 4 and 6 months of age than since‐birth exclusive breastfeeding, measured by reviewing all daily records since birth (Aarts et al., 2000). The discrepancy resulted from the high proportion of infants who had been given water or other liquids at some point since birth, but not on the previous day (Aarts et al., 2000).

A since‐birth recall (EBF‐SB) among infants 0–5.9 months of age may still overestimate the proportion of infants that are exclusively breastfed for the entire recommended period because the implicit assumption when using this measure remains—that infants’ feeding practices at the time of the survey will continue until they are 6 months old. In countries where prelacteal feeding is common, the since‐birth recall may result in low estimates of exclusive breastfeeding for 6 months, by eliminating from the numerator newborns who received prelacteal feeds but then were exclusively breast fed for the remainder of the 6 month period. It is important to note that the methods described here were investigated as alternatives to the EBF‐24H indicator currently recommended by WHO and UNICEF. In addition to measuring the proportion of infants exclusively breastfed for entire recommended period, it is also important to measure the proportion of infants who receive prelacteal feeds. In 2021, in addition to EBF‐24H, the WHO and UNICEF incorporated the following indicator for assessing infant and young child feeding practices, 'Percentage of children born in the last 24 months who were fed exclusively with breast milk for the first two days after birth', referred to as EBF2D (WHO & UNICEF, 2021). This offers a way to contextualise feeding practices and to explain what these measures tell us about the population, overall. One review of breastfeeding recall, which included studies from low‐, middle‐ and high‐income countries, concluded that recall of initiation and any duration of breastfeeding is valid and reliable for recall periods less than 3 years, though recall of introduction of foods and liquids is less reliable (Li et al., 2005). In India, estimating EBF by asking mothers of infants in their 9th month about the age at which 13 foods and liquids were introduced (using a calendar) performed well when validated against a prospective measure of EBF (Agampodi et al., 2011). A more recent study from Brazil showed that, compared to a 24‐h recall at 3 months of age, measurement of exclusive breastfeeding duration at 12 months of age by asking about the age at which specific foods and liquids were introduced provides a valid measure of exclusive breastfeeding duration (Schneider et al., 2020). Though the latter suggests that the prevalence of exclusive breastfeeding for the full 6‐month period could be estimated by obtaining retrospective data from children 6–11.9 months, this may not be true for longer recall periods.

Table 2 describes the advantages and disadvantages of each method explored in terms of accuracy, feasibility, data availability and alignment with the global recommendation for practice.

**Table 2:** Advantages and disadvantages, by method

Method	Advantages	Disadvantages
EBF‐24H Prevalence of exclusive breastfeeding among infants less than 6 months, based on a 24‐h recall	Easy to collect Short recall period results in more accurate reporting Data are readily available for many countries and across many time points	Overestimates the proportion of infants exclusively breastfed for 6 months
EBF‐SB Percentage of infants less than 6 months who were not given anything other than breast milk since birth	Easy to collect Excludes from the numerator infants who may have consumed only breast milk on the previous day, but something other than breast milk before that thereby reducing the extent to which this indicator overestimates the proportion of children exclusively breastfed for the first 6 months	This type of recall is not usually collected in large‐scale surveys so data are not readily available May overestimate the proportion of infants exclusively breastfed for 6 months Unless specifically probed for, prelacteal feeds may not be reported
EBF‐AI Percentage of infants 6–11.9 months who did not consume anything other than breast milk for their first 6 months of life	Easy to collect Only includes in the numerator children who were exclusively breastfed for 6 months, aligning with the WHO recommendation	Longer recall period may be less accurate This type of recall is not usually collected in large‐scale surveys so data are not readily available Responses may be subject to heaping
Pullum's method of estimating exclusive breastfeeding at 6 months of age, using either a 24‐h or a since‐birth recall (EBF‐24H‐Pul and EBF‐SB‐Pul)	Simple to apply The EBF‐24H‐Pul can be calculated using existing data collected for EBF‐24H, which is widely available	Sample sizes for the two narrow age bands used for this method may be small in some surveys

Abbreviations: EBF‐AI, age at introduction; EBF‐SB, since‐birth recall; EBF‐SB‐Pul, Pullum's method applied to a since‐birth recall; EBF‐24H, 24‐hour recall; EBF‐24H‐Pul: Pullum's method applied to a 24‐hour recall; WHO, World Health Organization.

The method proposed by Pullum (2014) is simple to apply and approximates the percentage of infants who exclusively breastfeed for 6 months, aligning the indicator with the WHO recommendation. An advantage of this method is that it is possible to construct the indicator using existing data from large‐scale household surveys such as the DHS and the UNICEF MICS. A limitation of the method is that calculating prevalence estimates may not always be possible in surveys that do not specifically sample infants in the required age ranges (i.e., 4–5.9 months of age and 6–7.9 months of age). Large household surveys that collect data on a variety of topics, such as the DHS and MICS, often use samples that are representative of women aged 15–49, only a small portion of which have infants in the 0–5.9 month age range. In these surveys, calculating EBF‐24H is done using a subsample. The method proposed by Pullum requires calculating EBF separately in two, 2‐month age bands, which further reduces the subsample. This limitation is easily overcome in surveys that are designed to collect data specifically about infants. While the EBF‐AI measure would not suffer from this sampling challenge, the data to construct that indicator are not usually included in existing large‐scale household surveys.

One limitation of the EBF‐AI estimate is that it represents a prevalence of 6 months of exclusive breastfeeding at a point in time that is, on average, 3 months before the survey. This compromises the comparability between the EBF‐AI and the two‐point prevalence indicators (EBF‐24H and EBF‐SB) when collected in the same cross‐sectional survey. In contexts where breastfeeding practices are changing rapidly (e.g., where there is intense breastfeeding promotion or following a natural disaster or other emergency), the EBF‐AI measure would be expected to be different from the estimates obtained using 24‐h recall or since‐birth recall among infants 0–5.9 months of age in the same survey. Limiting our analysis to baseline data was intended to mitigate this limitation, knowing that these data were collected before a period of intense breastfeeding promotion. Another limitation of the EBF‐AI estimate is that the responses to these questions may be subject to heaping; for example, mothers respond in whole months, rather than precise ages when asked about the introduction of foods and liquids. In the three data sets used for this study, the age at introduction was recorded in whole months, so it was not possible to analyse heaping at whole months. The use of a calendar to obtain the duration of any breastfeeding was shown to reduce heaping at reported ages of 6, 12, 18 and 24 months (Becker & Diop‐Sidibé, 2003). When collecting retrospective data from infants in their 9th month, the use of a calendar to obtain the age at which foods and liquids were introduced performed better than maternal recall of duration of EBF when these two methods were compared to prospective data (Agampodi et al., 2011). The use of a calendar to collect EBF‐AI may improve the quality of reporting.

A limitation of these analyses is that all of the methods rely on mothers’ self‐report, which is subject to recall, social desirability and other biases. None of these survey‐derived methods represent a gold standard for measuring exclusive breastfeeding and it is impossible to assess from these data whether one method is more accurate than another. However, we show differences in estimates and discuss the likelihood of accuracy in addition to alignment with the WHO recommendation to exclusively breastfeed for 6 months and the feasibility of data collection.

Currently, only an estimated 44% of infants worldwide exclusively breastfeed within the first 6 months of life, which is derived using the EBF‐24H measure, as recommended by the WHO (UNICEF, 2021; WHO, n.d.a). While the Global Nutrition Target aims to increase the rate of exclusive breastfeeding up to 50% among all infants 0−5.9 months, even if this target is achieved, it is likely that the proportion of infants who exclusively breastfeed for the full 6 months as recommended, could be much lower, as these results demonstrate (WHO, n.d.b). While it is true that the target of 50% may have been set at a different level if a different measure had been used, the fact remains that even if the current target is met, the majority of infants will not have received the benefits of 6 months of exclusive breastfeeding. Regardless of the measure, to truly achieve that goal, breastfeeding‐supportive policies and increased investments in equitable coverage of programmes, like integrating the Ten Steps to Successful Breastfeeding into National Standards of Care, community‐based peer and family support, education, community mobilisation, social marketing and paid parental leave policies are still crucially needed (Cresswell et al., 2019; Kavle et al., 2017; Nguyen et al., 2017; Sanghvi et al., 2016; USAID, IYCN and Carolina Global Breastfeeding Institute 2012; WHO & UNICEF, 2019). Further research on and evaluations of breastfeeding interventions using a variety of measures for EBF could help policy makers and other stakeholders better understand gaps and target programmes to support families in reaching the WHO recommendations for EBF.

## 5 CONCLUSION

Our analyses show measurement‐based differences in exclusive breastfeeding prevalence estimates calculated from the same datasets. Each method has pros and cons in terms of accuracy, feasibility, data availability and alignment with the global recommendation for practice. Different survey‐based methods for estimating the proportion of infants who are exclusively breastfed for 6 months should be studied further when accurate prevalence estimates are required, for example, to inform programme and policy decisions. Careful communication and dissemination of findings regarding alternative methods are important to avoid confusion, particularly with key decision makers and other stakeholders. Our analysis suggests that feasible, easy‐to‐collect alternatives to the widely used 24‐h recall method may exist to provide estimates that more closely align with the WHO recommendation of 6 months of exclusive breastfeeding. Exclusive breastfeeding prevalence estimated retrospectively from infants 6–11.9 months was higher than those estimated using Pullum's weighted average method. Because data are widely available and many countries have multiple data points, and it is the basis for the Global Nutrition Target, the EBF‐24H indicator will remain useful to assess trends. Meanwhile, continuing to develop easy‐to‐collect, survey‐based methods that more accurately measure 6 months of exclusive breastfeeding is important. Additional validation of some of the methods described here, namely, the EBF‐AI and Pullum's method, using prospective birth cohorts followed through panel studies with frequent recalls, observations or biological samples can be used to identify and test survey‐based methods that would be as feasible to collect in large‐scale surveys as the EBF‐24H, and could more accurately align with and measure the WHO exclusive breastfeeding recommendation.

## AUTHOR CONTRIBUTIONS

*Study conception and design*: Silvia Alayón and Jennifer Yourkavitch. *Data analysis*: Silvia Alayón, Veronica Varela, and Jennifer Yourkavitch. *Interpretation of results*: Silvia Alayón, Altrena Mukuria‐Ashe, Jeniece Alvey, Sarah Pedersen, Erin Milner, Veronica Varela, and Jennifer Yourkavitch. *Draft manuscript preparation*: Silvia Alayón. All authors reviewed the results and approved the final version of the manuscript.

## CONFLICTS OF INTEREST

Erin Milner, Jeniece Alvey, and Sarah Pedersen are employed through the USAID funded Global Health Technical Professionals (GHTP) and Sustaining Technical and Analytical Resources (STAR) mechanisms and are employed by one of the implementers, The Public Health Institute. The opinions herein are those of the authors and do not necessarily reflect the views of the U.S. Agency for International Development or the U.S. Government, or the Public Health Institute.

## Data Availability

The data that support the findings of this study are available in Dataverse. These data were derived from the following resources available in the public domain. See URLs below. Bangladesh: 10.7910/DVN/FO8WDU Nigeria: 10.7910/DVN/ZI1HUO Viet Nam: 10.7910/DVN/AORZAU
